# Assessing Vehicle Wandering Effects on the Accuracy of Weigh-in-Motion Measurement Based on In-Pavement Fiber Bragg Sensors through a Hybrid Sensor-Camera System

**DOI:** 10.3390/s23218707

**Published:** 2023-10-25

**Authors:** Xinyi Yang, Xingyu Wang, Joseph Podolsky, Ying Huang, Pan Lu

**Affiliations:** 1 Department of Civil and Environmental Engineering, North Dakota State University, Fargo, ND 58102, USA; xinyi.yang@ndsu.edu (X.Y.); xingyu.wang@ndsu.edu (X.W.); 2 Minnesota Department of Transportation, Office of Materials and Road Research, Maplewood, MN 55109, USA; joseph.podolsky@state.mn.us; 3 Department of Transportation, Logistics and Finance, North Dakota State University, Fargo, ND 58102, USA; pan.lu@ndsu.edu

**Keywords:** weigh-in-motion, Fiber Bragg Grating sensor, image process, hot mix asphalt

## Abstract

Weigh-in-motion (WIM) systems are essential for efficient transportation and monitoring parameters such as vehicle number, speed, and weight to ensure regulatory compliance and enhance road safety. Recently, WIM measurements using the Glass Fiber Reinforced Polymer Fiber Bragg Grating (GFRP-FBG) sensors have shown robustness and effectiveness. However, the accuracy of weight evaluation using the WIM systems based on GFRP-FBG sensors can be significantly influenced by the vehicle-wandering effect, which introduces uncertainties in wheel position determination and weight calculations. This paper assessed the impact of vehicle wandering on the accuracy of a WIM measurement system based on GFRP-FBG sensors by utilizing a new hybrid sensor-camera system that integrates roadside cameras and in-pavement GFRP-FBG sensors. The detailed methodology and framework of the developed hybrid system are introduced, followed by field testing on Highway I-94 in the United States. The field testing results indicate that by using the hybrid system, the wheel load detection accuracy of the WIM system based on GFRP-FBG sensors can be controlled to be a Type I or Type III WIM according to the ASTM 1318E-09 standard, with an average accuracy ranging from 87.83% to 94.65%. At the same time, when the wander distance is less than or equal to 9 cm, the developed WIM system proves to be very cost-effective as it only comprises two GFRP-FBG sensors, one temperature FBG sensor, and one camera. These findings indicate the practical potential to enhance the accuracy of WIM systems based on GFRP-FBG sensors designed for highways for low-coast, reliable, and accurate measurements by addressing vehicle wandering effects.

## 1. Introduction

The number of registered vehicles in the United States continues to rise, with a reported total of approximately 282 million in 2021 [1], which increases concerns regarding road maintenance and safety as they are closely tied to traffic data, such as traffic volume and vehicle weight. The field of urban traffic data collection offers various methods, including data from swiping transit cards, online ride-hailing services, and bikesharing usage [2,3,4]. For highway traffic, the primary traffic data collection methods encompass the automatic traffic recorder and weigh-in-motion (WIM) systems; however, the automatic traffic recorder can only provide vehicle counts, while WIM collects detailed data on vehicles speed, axle load, gross vehicle weight, and classification.

Weigh-in-motion systems have become integral to modern transportation infrastructure, playing a multifaceted role with diverse applications. In the field of traffic management, WIM systems contribute to optimizing traffic flow, reducing congestion, and supporting informed decision-making for road improvements. WIM technology also aids in safety enforcement and environmental protection. It helps monitor weight limits, ensures compliance with regulations, and guards against the detrimental effects of overloaded vehicles, enhancing road safety while reducing maintenance costs and environmental impact. They provide crucial data for pavement design, helping engineers create roads capable of withstanding the heavy loads of modern vehicles [5]. However, the accuracy of WIM systems requires careful consideration, given that various factors can influence WIM accuracy, including driving speed, weather conditions, and road roughness [6,7,8]. In general, two primary data acquisition methods for practical WIM systems are utilized, including the embedded sensor system and the image capture system. The sensor system integrates specialized sensors into the road surface, detecting vehicle forces to calculate real-time weights. Conversely, the image-capturing system strategically positions cameras to estimate weight by analyzing the tire’s figure as the vehicle moves.

For a WIM system based on in-pavement sensors, the embedded sensor system consists of two main components: a data acquisition system and sensors integrated into the road surface. The sensors commonly used for this purpose include bending plates, load cell plates, polymer piezoelectric sensors, quartz piezoelectric sensors, and strain gauge strip sensors [9]. However, these electrical sensors, such as bending plates, load cell plates, polymer piezoelectric sensors, and quartz piezoelectric sensors, tend to be highly susceptible to their surrounding environment, which often leads to issues such as electromagnetic interference, relatively short lifespans, and moderate levels of measurement errors [10]. To overcome these challenges, Fiber Bragg Grating (FBG) technology has emerged as an attractive alternative for WIM systems based on in-pavement sensors. FBG technology offers several advantages, including immunity to electromagnetic radiation, non-conductive nature, lightweight design, spark-free operation, intrinsic safety, high sensitivity to strain, and embedding compatibility with structural elements [11]. FBG sensors find widespread application in research to measure parameters like strain, stress, temperature, pressure, and vibrations across diverse fields, encompassing structural health monitoring [12,13], aerospace [14], etc. However, it is worth noting that bare FBG sensors are quite delicate and can be easily damaged when exposed to harsh and demanding environments. To address this, the proper packaging method of the FBG sensors becomes essential [15]. Recently, Glass Fiber Reinforced Polymer (GFRP) has been demonstrated to be an effective choice for packaging FBG sensors for WIM systems in asphalt pavements to detect real-time wheel weights due to its impressive strength, durability, resistance to corrosion, and ability to withstand fatigue, known as GFRP-FBG sensors [10,16].

However, due to the fact that the GFRP-FBG sensors are point sensors, the accuracy of GFRP-FBG sensors for WIM measurement can be significantly influenced by the occurrence of the wandering effect, stemming from the disparity between sensor placement and actual wheel load positions [10]. This mismatch often results in inaccuracies as the sensors are fixed in location, failing to precisely align with the varying wheel positions of passing vehicles. As a consequence, this phenomenon introduces uncertainties and inconsistencies in weight measurements, undermining the reliability of the collected data. This difficulty arises from factors such as sensor accuracy, weather conditions, and road surface smoothness associated with current techniques [17]. Therefore, alternative approaches must be explored to enhance the accuracy of the WIM system based on FBG sensors, more specifically, the GFRP-FBG sensors.

On the other hand, a multitude of cutting-edge research studies have emerged, investigating pioneering approaches like computer vision within the domain of WIM systems without the need to install sensors beneath the road surface. Feng et al. [18] introduced a novel approach by leveraging computer vision methods to analyze images of moving vehicles. In this study, the researchers extracted tire deformation parameters, including tire-roadway contact length, vertical deflection, and tire make/model identification. They retrieved tire width and manufacturer-recommended tire inflation pressure from a tire information database and calculated vehicle weight by totaling the products of each tire’s contact area and inflation pressure [18]. Furthermore, Kong et al. [19] employed cameras to capture images of moving vehicles and subsequently utilized computer vision techniques to detect tire-road contact areas and inflation pressure, achieving non-contact WIM solutions. The primary advantage of those studies lies in the image analysis of moving vehicles to estimate vehicle weight, where the images are captured by cameras placed near the road, eliminating the need for sensor installation. However, a limitation arises from the use of manufacturer-provided inflation pressure, which may not accurately reflect the actual tire conditions. Variations in contact pressure can occur due to tire type, tire load, and inflation pressure, ranging from significantly less than the inflation pressure to up to 10–30% more [20,21,22,23]. Additionally, temperature fluctuations can lead to changes in inflation pressure [24]. This discrepancy is particularly pronounced for trucks carrying heavy loads, as the actual contact pressure may significantly differ from the manufacturer’s recommended inflation pressure.

Therefore, a WIM system solely using embedded FBG sensors or image-capturing systems exhibits specific limitations. The embedded sensor systems have concerns regarding the wandering effect, whereas the image-capturing systems encounter challenges in accurately measuring inflation pressure. However, these systems also offer notable advantages; for instance, the embedded sensor systems provide real-time weight calculations, while the image-capturing systems excel at capturing vehicle information.

To leverage the collective strengths of these systems and address their respective limitations, this study proposes a new hybrid WIM system combining embedded sensor systems utilizing in-pavement GFRP-FBG sensors and computer vision. This proposed approach aims to address the wandering effect in embedded GFRP-FBG sensor systems and achieve more accurate and comprehensive weight data by incorporating image-capturing systems. To achieve such a goal, this study developed a low-cost WIM system that integrated two GFRP-FBG sensors, a temperature compensation sensor, and a roadside camera. The GFRP-FBG sensors correlate the wheel loads with the strain measurements from the sensors; the temperature sensor compensates for the temperature effect; and the roadside camera pinpoints the wheel load location for assessing the wandering effect. Therefore, a meticulous calibration of the wandering effect was conducted by merging strain data extracted from GFRP-FBG sensors and the wheel load location captured through camera images. The methodology and framework for the proposed system are detailed, and the calibration process was facilitated on Highway I94 in the United States through the utilization of the KENPAVE software. With the developed hybrid system, the accuracy of WIM measurements using GFRP-FBG sensors has significantly improved to be applicable for Type I or Type III WIM systems according to the ASTM 1318E-09 standard [25] in a cost-effective manner and shows the great potential for real-time vehicle wheel load detection in transportation, road maintenance, and weight regulation compliance across research and industry.

## 2. Methodology

### 2.1. Framework for Weigh-in-Motion Using Camera Data and GFRP-FBG Sensors Fusion

**Figure 1:**
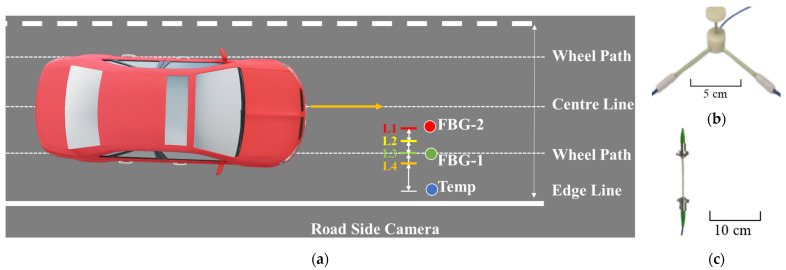
Photo of the (**a**) practical layout of the hybrid WIM system; (**b**) 3D; and (**c**) 1D GFRP-FBG sensors [26].

The layout of the proposed hybrid WIM system, depicted in Figure 1a, involves the utilization of a camera, two GFRP-FBG sensors, and one temperature compensation sensor. Either three-dimensional (3D) or one-dimensional (1D) GFRP-FBG sensors, as shown in Figure 1b,c [26], can be used. In the case of the 3D GFRP-FBG sensor, it integrates three distinct components for data collection along vertical, longitudinal, and transverse directions. The longitudinal component aligns parallel to the wheel path. The transverse component is perpendicular to it, and a vertical dimension extends to point at the asphalt surface. Based on a previous study [26], the longitudinal, transverse, and vertical segments possessed dimensions of 4.064, 4.064, and 3.048 cm, respectively, with an FBG positioned at the center of each component. Among a 3D GFRP-FBG sensor, the vertical component detects proximity but faces alignment challenges with passing vehicles, while the transverse aspect depends less on the matrix and requires a reasonable wheel load radius [10]. Conversely, the longitudinal part shows high sensitivity, less matrix independence, and an ample detection range, making it ideal for WIM measurements along the wheel path. Hence, the longitudinal component is usually chosen for evaluating high-speed WIM feasibility after field assessment. For an 1D GFRP-FBG, it can be positioned strategically in the longitudinal direction for wheel load detection.

**Figure 2:**
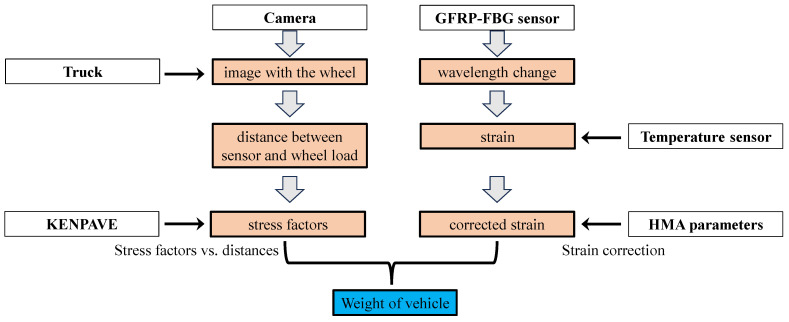
Integrated hybrid WIM system for accurate wheel load measurements.

Figure 2 further shows the network of the hybrid WIM system, which combines an embedded sensor system utilizing in-pavement GFRP-FBG sensors with computer vision through a camera. The camera captures images of the moving vehicle, with a particular focus on the vehicle’s wheels. This enables the system to calculate the distance between the sensor and the wheel loading point, which is crucial for KENPAVE’s analysis. KENPAVE software is employed to compute stress factors, aiding in the understanding of pavement behavior under changing loading positions and environmental conditions. These stress factors are influenced by sensor installation depth and distance from the loading point, which play a significant role in the comprehensive analysis of the wander effect’s impact. As the vehicle passes over the installed GFRP-FBG sensor, variations in wavelength occur due to temperature and strain changes. Following temperature compensation via a temperature sensor, the strain induced by the vehicle’s passage can be computed after including the HMA parameter. Then, after combining the results through the hybrid WIM system, the weight of the vehicle can be determined.

### 2.2. Wheel Load Measurement by GFRP-FBG Sensor

For the objectives of this study, the determination of vehicle weight is predicated on the utilization of two GFRP-FBG sensors, either the longitudinal segment of an 3D sensor or an 1D sensor, while the temperature sensor (T) is used in temperature compensation. As shown in Figure 1, specifically, all sensors can be installed on the right, left, or both sides (if there is enough budget) of the center line within a typical width of the driving lane. FBG-1 needs to be strategically placed above the wheel path, while FBG-2 can be located within a reasonable distance of FBG-1, which is 0.521 m away from FBG-1, as in the example in Figure 1a. Moreover, FBG-1, FBG-2, and the temperature sensors should be aligned on the same axis. To calibrate the positioning of both the actual elements and the figures, four specific positions are designated in this study. The positions L1 and L3 correspond to the placement of the FBG-1 and FBG-2 sensors, respectively. The L2 location is positioned equidistant between L1 and L3, and Location L4 is situated at a certain distance away from L3. Based on the layout of the GFRP-FBG sensors, this section further details how to measure wheel load from the GFRP-FBG sensors accurately using the hybrid system.

#### 2.2.1. Strain Collection by GFRP-FBG Sensor

For the FBG sensor, a portion of an optical fiber is exposed to an UV laser, and the optical FBG is made by laterally exposing the core of a single-mode fiber to a periodic pattern of intense ultraviolet light [9,27]. The specific wavelength of light (Bragg wavelength) will be reflected by the grating when a broadband incident light is transmitted into the fiber [9]. The Bragg wavelength can be exposed as [27,28]:

(1) λ=2nΛ

where n is the effective index of refraction and Λ is the grating periodicity of the FBG.

For the embedded FBG sensors, the measured field interacts with the sensitive optical fiber, causing the wavelength change of the transmitted light in the optical fiber [29,30]. Once vehicles pass through the GFRP-FBG sensors, the Bragg wavelength changes as a function of temperature and strain, which is dependent on the grating period [27]. The general expression of the strain–temperature relationship for the GFRP-FBG strain sensor and temperature compensation sensor can be described as [28]:

(2) $$\frac{∆\lambda }{\lambda }=\frac{∆\lambda_{\varepsilon }}{\lambda_{\varepsilon }}+\frac{∆\lambda_{T_{e}}}{\lambda_{T_{e}}}=\left(1-P_{e}\right)\varepsilon +(\alpha +\gamma )∆T_{e}$$

(3) $$\frac{∆\lambda_{T_{e}}}{\lambda_{T_{e}}}=(\alpha +\gamma )∆T_{e}$$

in which λ is the Bragg wavelength of the grating, which experiences strain and temperature changes, and where $\lambda_{T_{e}}$ and $\lambda_{\varepsilon }$ is Bragg wavelength of the grating, which only experiences temperature and strain change. The $∆\lambda_{T_{e}}$ and $∆\lambda_{\varepsilon }$ represent the alteration in Bragg wavelength due to temperature and strain, respectively. α, γ, and $P_{e}$ are the thermal expansion coefficient, thermal-optics coefficient, and optical elasticity coefficient, respectively. Then, the strain of the sensor can be determined as follows:

(4) $$\varepsilon =\frac{1}{\left(1-P_{e}\right)}(\frac{∆\lambda }{\lambda }-\frac{∆\lambda_{T_{e}}}{\lambda_{T_{e}}})$$

#### 2.2.2. Strain Correction Based on the Host Material

In addition, for the GFRP-FBG sensor, the optical fiber is covered by the protecting layer (GFRP) and host material (HMA). When the host material is different, the strain transfer error of the GFRP-FBG sensor is also different. Accounting for the impact of the packaging layer between the optical fiber and host material, the relationship between the average strain experienced by the GFRP-FBG sensor (${\bar{\varepsilon }}_{c}$) and the actual strain of the host material (${\bar{\varepsilon }}_{h}$) is as follows [31]:

(5) $${\bar{\varepsilon }}_{h}=\frac{{\bar{\varepsilon }}_{c}}{1-\phi }={k\bar{\varepsilon }}_{c}$$

where k is the strain transfer error modification coefficient and ϕ  is the measurement error of the packaged GFRP-FBG, which can be computed as [27]:

(6) $$\phi =\left|\frac{{\bar{\varepsilon }}_{c}-{\bar{\varepsilon }}_{h}}{{\bar{\varepsilon }}_{h}}\right|=\left|\frac{\cosh\left(\xi l_{f}\right)-1}{\xi l_{f}sinh(\xi l_{f})}\right|$$

where $l_{f}$ is the gage length of the package GFRP-FBG sensor and the ξ represents the eigen value of its characteristic function, which can be calculated as [31]:

(7) $$\xi^{2}=\frac{2}{E_{0}{r_{0}}^{2}(\left(\frac{1}{G_{GFRP}}\right)ln\left(\frac{r_{GFRP}}{r_{0}}\right)+\left(\frac{1}{G_{h}}\right)ln\left(\frac{r_{h}}{r_{GFRP}}\right))}$$

where $E_{0}$ and $r_{0}$ are the elastic modulus and outer radius of the optical fiber; $G_{GFRP}$ and $r_{GFRP}$ are the shear modulus and outer radius of the protecting layer (GFRP); $G_{h}$ and $r_{h}$ is the shear modulus and outer radius of the host material layer.

For different host materials, the shear modulus ($G_{h}$) is different, with the dynamic modulus (E) changed. The shear modulus at various temperatures and loading frequencies was generated using a constant Poisson’s ratio (μ) with the following equation [32]:

(8) $$G_{h}=\frac{E}{2(1+\mu )}$$

The dynamic measurement error is used to correct the strain values generated by the GFRP-FBG sensors based on different temperatures and loading frequencies, where the temperature is varied by seasons and the loading frequency is influenced by the contact radius of the tire.

For enhanced accuracy in strain values, the dynamic modulus of the material covering the sensor at different temperatures and frequencies is employed to calculate the measurement error between the collected and actual strain values. The dynamic modulus of elasticity and parameters, like the asphalt concrete pavement’s thickness and Poisson’s ratio for each road layer, are determined through laboratory testing. These data are then fed into the KENPAVE software, which generates the stress factor of the road at various locations and depths. Subsequently, the wheel load can be calculated using Equation (9).

(9) $$F=\frac{\varepsilon_{z}\;*\;E\;*\;a}{\alpha_{z}-v(\alpha_{r}+\alpha_{t})}$$

where $\varepsilon_{z}$ is the vertical strain that can be calculated through the FBG sensor and temperature sensor, E is the dynamic modulus of elasticity, a is the contact area, v is the Poisson ratio, $\alpha_{z}$, $\alpha_{r}$, and $\alpha_{t}$ is the vertical, radial, and tangential stress factor.

### 2.3. Integration of Image-Based Distance Determination and KENPAVE Analysis to Determine the Stress Factor

Given the fixed-sensor position, variations arise in the signal received by the FBG sensor as vehicles with varying wheel loading positions pass. To achieve precise weigh-in-motion data, knowing the wheel-loading positions is paramount. Additionally, accounting for the stress factor based on road parameters and vehicle characteristics becomes crucial in relation to the distance between the wheel loading position and the sensor. Hence, this section introduces methodology for determining wheel-load positions from the figures featuring the wheels and elucidates the process of calculating the stress factor utilizing the KENPAVE software.

#### 2.3.1. Determining Wheel Load Position Using Camera-Captured Figures

**Figure 3:**
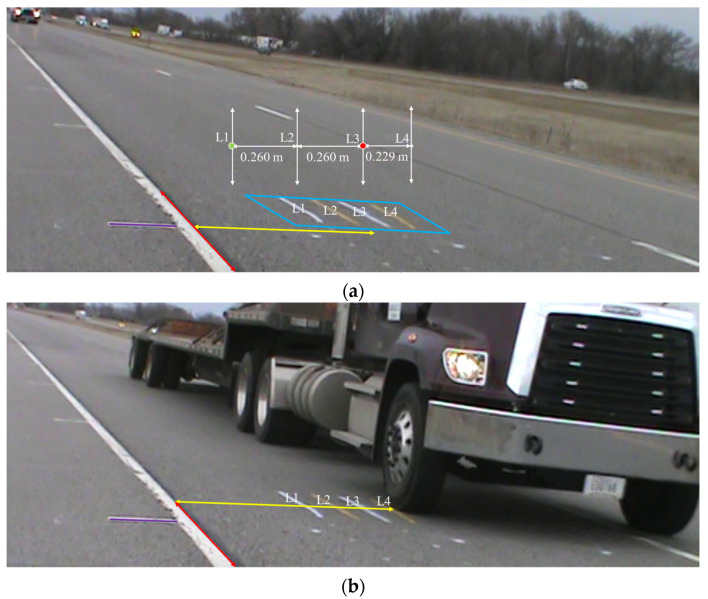
Accurate wheel load position determination and calibration process (**a**) calibration area with marked and calibrated lines; (**b**) example of a vehicle passing the calibration area.

Accurately determining the wheel load position is crucial for WIM due to the fixed sensor positions. The signals generated as a vehicle passes over the sensors vary based on the wheel load position. Four positions are identified in this process, with two located at the sensor locations (L1 and L3) and the other two adjacent. For example, L2 can be situated at a distance of 26 cm from each sensor location, and L4 can be at a distance of 22.9 cm from L3, respectively. These positions are marked by lines, as illustrated in Figure 3. To ensure safety and avoid direct positioning in front of moving vehicles, the cameras need to be placed right ahead of the vehicle on the roadside.

Despite the camera being properly set up and capturing images with the wheels, an issue arises in accurately determining the precise distance between the wheel loading position and the embedded sensor through the image due to visual perspective. Despite distance appearing shorter and the potential for actual distances to remain consistent, this issue persists due to a visual distortion arising from the image’s perspective, where objects positions farther away seem compressed, resulting in them appearing shorter even though their true distances remain unaltered. Within this study, the four marked lines (for locations L1 to L4), in conjunction with three calibration lines, hold a pivotal role in quantifying the distance between the wheel loading position and the embedded sensor. In scenarios where no vehicles are present, the four marked lines and three calibration lines are shown in Figure 3a. The known distance between the edge line and the four marked line traces on the road formed a pivotal aspect in defining the wheel’s location. This position is determined through the utilization of three calibration lines:

1. Purple calibration line: White lines usually exist on the side of the highway edge line, which is used as the purple calibration line, deliberately positioned consistently above the same spot and meant to align with the white line. If the purple calibration line fails to align with the white line, it signifies the potential for camera position shifts caused by windy conditions, leading to the possibility of inaccurate determinations of locations;
2. Red calibration line: The highway edge lines (red line) can be used as another reference as they determine the wheel loading position of trucks in the direction parallel to the edge line;
3. Yellow calibration line: The lines perpendicular to the highway edge lines (yellow line) can be used to determine the wheel loading position of trucks in the direction vertical to the edge line.

As depicted in Figure 3a, once the camera positioning is confirmed using the purple line, the lengths of the yellow calibration lines and their corresponding red calibration lines are taken as input variables. These inputs are used to determine the output variable, which represents the actual distance between the edge line and the marked line. The trained model will then be employed to predict the wheel’s location, similar to the scenario depicted in Figure 3b, where the lengths of the yellow and red lines are inputted to determine the wheel’s position accurately.

#### 2.3.2. Stress Factor Determination Using KENPAVE Software

In the field of pavement analysis, when a vehicle’s wheels apply pressure to the road surface, resulting deformations manifest at varying depths and positions. Consequently, it becomes essential to determine the stress factor at different depths and distances from the loading point (determined by the wheel location in Section 2.3.1.), considering elements like contact area, dynamic modulus of the road, temperature, and loading frequency as the vehicle moves along the road.

To address this challenge, the KENPAVE software [33] is employed in this study, offering a solution to the wander effect and providing specialized tools extensively used in civil engineering and pavement analysis. In this study, KENPAVE facilitates the computation of stress factors, crucial for understanding pavement behavior under varying loading positions, temperatures, and environmental conditions. Notably, stress factors are influenced by parameters such as sensor installation depth, distance between the sensor, and loading position.

With all the requisite information (including dynamic modulus, Poisson’s ratio, and thickness of each road layer at specific temperatures and loading frequencies from the lab) ready for input into KENPAVE, the only missing elements are the contact area and dynamic modulus. Based on the research that compared the real tire contact area (detected by vehicle drive through a paper with a record footprint of the wheel) with the measured contact area by different equations, including rectangular, circular, deflection, and oval [19,34,35], the contact area measured by the oval method generated the lowest average error [19]. The equation to evaluate the contact area based on the oval method is [19]:

(10) $$A=\frac{2}{3}\;*\;l\;*\;w+\frac{\pi }{3}\;*\;\frac{l}{2}\;*\;\frac{w}{2}$$

where l and w are length and width of the contact area.

On the other hand, for different pavement roads, the dynamic modulus was different, and it was influenced by the temperature and loading frequency. Using dynamic modulus data collected in the field and the NCAT of the HMA in the field experiments, a dynamic modulus master curve can be developed by shifting twenty-four results gained through testing at different temperature-frequency combinations using the following equations [36,37]:

(11) $$\log\left|E\right|=\delta +\frac{Max-\delta }{1+e^{\beta +\gamma \;*\;log\omega_{r}}}$$

where E  and Max are dynamic modulus and limiting maximum modulus (6.895 GPa [38]); δ, β, and γ are fitting parameters. The logarithm of the reduced frequency at the reference temperature ($\omega_{r}$) can be computed based on the Arrhenius equation [36,39]:

(12) $$log\omega_{r}=log\omega +\frac{\Delta E_{a}}{19.14714}(\frac{1}{T}-\frac{1}{T_{r}})$$

where ω is the loading frequency at the test temperature. $T_{r}$ and T are the reference temperature and test temperature (K°) with the test temperature determined using the installed temperature sensor situated next to the GFRP-FBG sensor. $\Delta E_{a}$ is the activation energy (treated as a fitting parameter).

Assuming the moving load only impacted the sensor within a loading area six times the contact radius (a) between the tires, the loading frequency can be estimated by vehicle speed divided by the travel length (12 times the contact radius) [10] as follows:

(13) $$\omega =\frac{v}{12a}$$

Once the contact area and dynamic modulus have been calculated, all necessary input data for the KENPAVE software is available, and the software can then generate stress values and subsequently calculate the stress factor for varying distances between the sensor and the wheel loading position. Considering that sensor positions can differ and even the same vehicle passing by with varying wheel load positions can result in different signals received by the sensor, this study aims to utilize the stress factor produced by the software for calibration purposes. This approach is taken to mitigate the impact of the wandering effect on the accuracy of WIM results.

## 3. Materials and Field Validation Testing

To validate the proposed hybrid WIM system, a field study was conducted on Interstate 94 westbound, located on the MnROAD mainline between exits 201 and 194 in Albertville, MN, USA, with sensors meticulously positioned just beneath the asphalt pavement road surface. This section provides an overview of the field experiment encompassing pertinent details about the field’s location, pavement characteristics, and the determination of dynamic modulus for the specific road segment. Additionally, it outlines the deployment of sensors, their positioning, and the methodological setup of the experiment.

### 3.1. Field Experiment Setup

**Figure 4:**
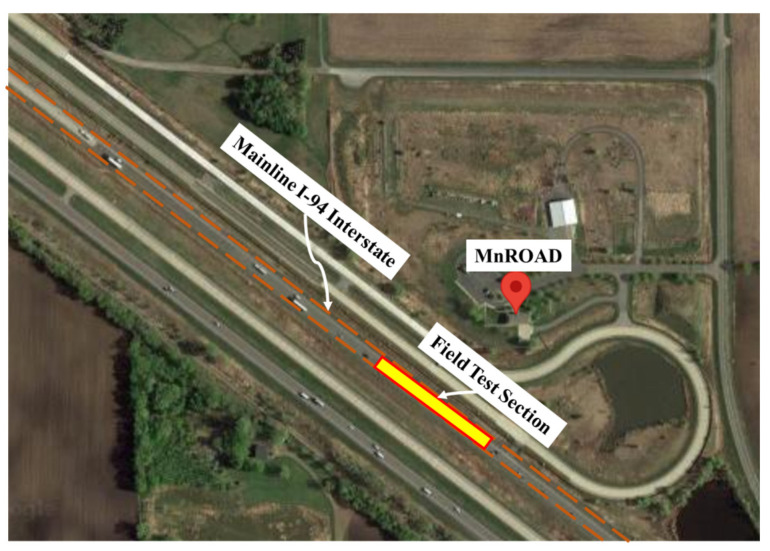
Test section location on MnROAD Mainline where the experiment was held.

The field tests took place on a designated road within the MnROAD research facility, an establishment dedicated to pavement research and equipped with a range of pavement materials. Administered by the Minnesota Department of Transportation, MnROAD encompasses several roadways that each contain several test sections, with the majority of test sections located on the MnROAD Mainline (3 miles of I-94 WB interstate), Old I-94 Westbound (3.5 miles of the original westbound I-94 built during the 1970s), and a 2.5-mile low-traffic volume test track. This extensive site boasts over 80 distinct test sections strategically situated along both the mainline and original westbound I-94 segments. The field test for this study was carried out on a specified test section, as highlighted in Figure 4.

**Figure 5:**
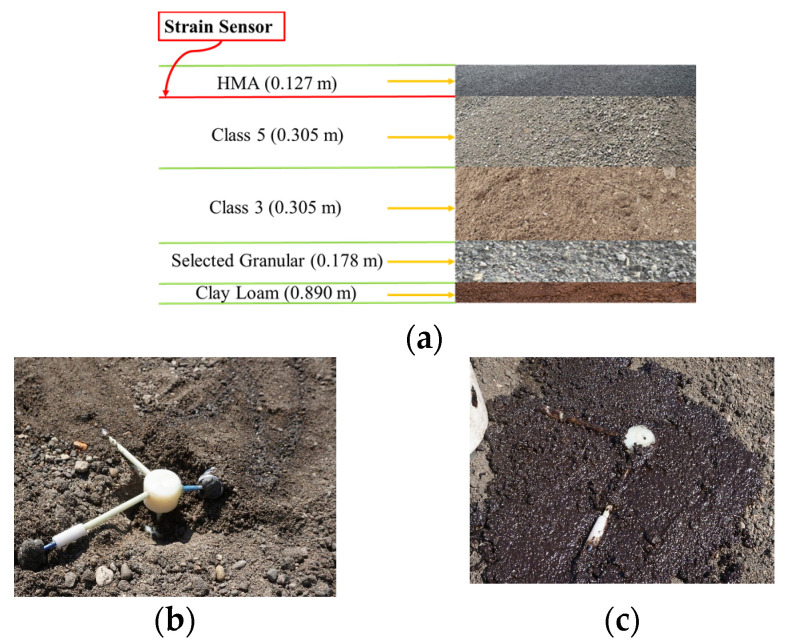
(**a**) Flexible pavement cross section of the test section; (**b**) GFRP-FBG sensor placement above MNDOT class 5 granular base; (**c**) safeguarding GFRP-FBG sensor placement with HMA layer.

The flexible pavement cross section of the test section is shown in Figure 5a and is composed of hot-mix asphalt (HMA), MnDOT Class 5 granular base, MnDOT Class 3 sub-base, selected granular layer, and clay loam [40]. The GFRP-FBG sensors were strategically positioned above the MnDOT Class 5 granular base, as illustrated in Figure 5b. To ensure the stability and integrity of their placement during subsequent construction stages, a thin layer of HMA was applied to gently secure the sensors in their designated locations. This safeguarding step was undertaken before the laying of the 0.127 m thick HMA layer, as shown in Figure 5c. This comprehensive approach guaranteed that the sensors remained in their intended positions throughout the pavement construction process.

Results from previous asphalt mixture performance testing experiments conducted by MnROAD and the National Center for Asphalt Technology, obtained from MnROAD, were utilized in this study. The testing involved two specimens with 150 mm diameter and 38 mm height (the same material as the warm mix asphalt layer of the test section), which were used in dynamic modulus testing. The dynamic modulus testing consisted of eight frequencies and three temperatures, which generated 24 temperature and frequency combinations. The average results from testing the two test specimens are shown in Table 1 [41].

### 3.2. Field Installed GFRP-FBG Sensors

The field sensor installation implementation followed Figure 1a. For the objectives of this study, the determination of vehicle weight was predicated on the utilization of the longitudinal segment of one 3D sensor (FBG-1) and one 1D sensor (FBG-2), while the temperature sensor was for temperature compensation. These sensors were meticulously positioned within the driving lane of the two-lane road on I-94. Specifically, all sensors were situated on the right side of the center line within the 3.658 m width of the driving lane. FBG-1 was placed above the wheel path, positioned 0.914 m away from the edge of the fog line, while FBG-2 was located 0.521 m away from FBG-1. Moreover, FBG-1, FBG-2, and the temperature sensors were aligned on the same axis. The positions L1 and L3 corresponded to the placement of the FBG-1 and FBG-2 sensors, respectively. The L2 location was positioned equidistant between L1 and L3, at a distance of 0.260 m from each. Location L4 was situated at a distance of 0.229 m from L3.

Previous research on the GFRP-FBG sensor [27], the elastic modulus ($E_{0})$ and outer radius ($r_{0}$) of the optical fiber, the shear modulus ($G_{GFRP}$) and outer radius ($r_{GFRP}$) of the packaging layer (GFRP), and the outer radius of the host material layer ($r_{h}$) are shown in Table 2. The gauge lengths of the longitudinal ($l_{f}$), transverse, and vertical components of the optical fiber were 70 mm, 70 mm, and 14 mm, respectively. The shear modulus of the host material layer (which is HMA in this study) was calculated based on the dynamic modulus of elasticity and Poisson’s ratio of the host material. This calculated shear modulus and the parameters depicted in the figure were then employed to calculate the measurement error attributable to the host material. This correction was subsequently applied to adjust the strain calculations obtained from the sensor.

### 3.3. Experimental Setup

The experimental design was targeted to evaluate vehicle performance at various positions with driving speeds of 16.093 km/h (10 mph), 32.187 km/h (20 mph), 40.280 km/h (30 mph), and 64.374 km/h (40 mph). A semi-truck passed over the two FBG sensors located at positions L1 and L3 (depicted in Figure 3). The same driver drove the truck over each position a total of seven times, aiming to align the wheels directly above the sensor positions.

However, ensuring precise control over the truck’s movement within the specific areas and at the designated speed presented a challenge, particularly when the two locations were in close proximity. Consequently, both the sensor data and captured images were employed to accurately calculate the vehicle’s exact speed and location as it passed through the specified points.

**Figure 6:**
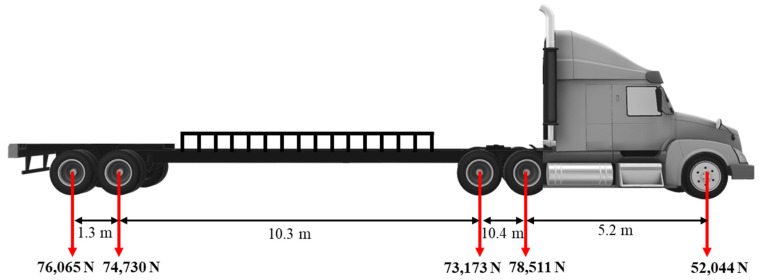
Layout of the semi-truck used in the MnROAD field test.

The field test utilized a semi-truck from the MnROAD facility, which had a total gross weight of 354,523 N. The vehicle configuration is illustrated in Figure 6. This study specifically concentrated on the weight measurement of the first axle equipped with a single tire.

## 4. Experimental Results and Discussion

The subsequent experimental results and discussion section highlight: (1) strain values from the same vehicle positioned differently during each drive; (2) a model trained to calculate distances between the wheel loading position and the edge line, aiding in determining the distance between the wheel loading position and the embedded sensor; and (3) stress factors for varying sensor-wheel distances. Through the application of strain and stress factors adjusted with distances derived from image analysis, the accuracy of wheel load assessment is evaluated.

### 4.1. Utilizing GFRP-FBG Sensors for Wavelength-Based Strain Calculation

**Figure 7:**
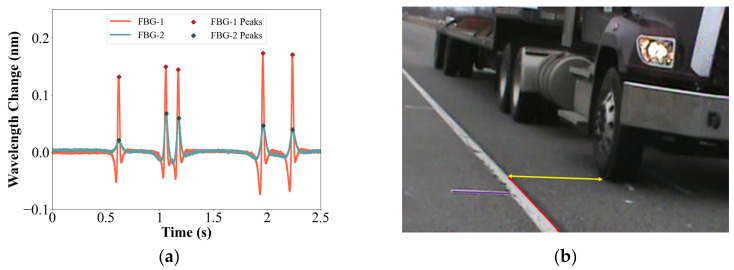
(**a**) Wavelength change generated by vehicle passage; (**b**) corresponding wheel loading position for FBG peaks.

Strain calculation was conducted using Equations (2)–(4), with the GFRP-FBG sensors collecting wavelength data and incorporating temperature compensation via a temperature sensor. As a vehicle passed over the sensors, a change in wavelength occurred, as illustrated in Figure 7a. In Figure 7b, the five wheels corresponded to the wavelength peak positions. In terms of the wheel location in Figure 7b, the wheel loading directly above the FBG-1 sensor was farther away from FBG-2. As a result, the wavelength changes and peaks recorded by FBG-1 were larger than those observed by FBG-2, as depicted in Figure 7a.

Given that accuracy in evaluating wheel load is higher when the wheel loading point is in proximity to the sensor, the data from FBG-1 was utilized for cases where the wheel loading point was near FBG-1, and similarly, the data from FBG-2 was employed when the wheel loading point was close to FBG-2. The collected wavelength change data from FBG-1, FBG-2, and the temperature sensor (T) for the 14 vehicle runs and the calculated strain derived from the wavelength change are presented in Table 3.

### 4.2. Calibration Line Utilization and Modeling for Distance Calculation

**Figure 8:**
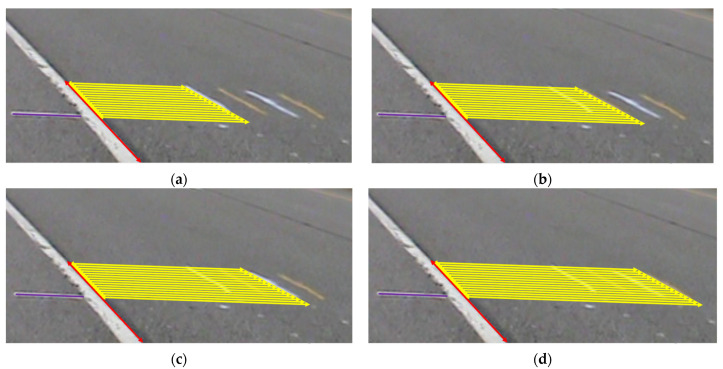
Calibration lines for location (**a**) L1; (**b**) L2; (**c**) L3; (**d**) L4.

With the purple calibration line consistently employed for determining the camera’s location, the four distinctive marked lines at locations L1, L2, L3, and L4 each used the same red calibration line, along with 15 yellow calibration lines (as shown in Figure 8), which differed for each location. The lengths of the red and yellow calibration lines, measured in meters, are presented in Table 4.

These measurements were employed to train a linear regression model as shown in Equation (14)—a fitted equation for calculating the distance from the edge line to the wheel location when inputting the lengths of the red ($l_{r}$) and yellow calibration lines ($l_{y})$. The model’s R-squared value and standard error are 0.9989 and 0.3668, respectively, when the confidence interval is set at 95%, indicating a good fit to the actual data.

(14) $$distance=-32.307+5.148\;*\;l_{r}+7.203l_{y}$$

The identical vehicle was intentionally driven over the sensors a total of 14 times, with seven of these passes deliberately executed near location 1 (FBG-1), and the remaining seven near location 2 (FBG-2). Subsequently, the lengths of the red and yellow calibration lines corresponding to the 14 vehicle runs were incorporated as input for the previously trained model. This model served to estimate the distance from the edge line to the wheel loading position. It’s important to clarify that the designated wheel loading position pertains to the right side of the wheel, not the midpoint, which signifies the actual wheel loading location. To rectify this discrepancy, the revised distance was corrected by adding half of the wheel width, equivalent to 0.103 m. Both the model-derived distances and the adjusted measurements are presented in Table 5 for comparison. In this table, a plus sign (+) indicates that the wheel loading point resided on the sensor side closer to the centerline, while a minus sign (−) denotes that the wheel loading point was situated on the sensor side nearer to the edge line. The sequence of vehicles was based on the absolute value of the revised distance derived from the FBG-1 and FBG-2 sensors.

### 4.3. Stress Analysis with KENPAVE Software in Pavement Structures

The KENPAVE software [33], taking into account various input parameters, including the number of pavement layers, the depth of the GFRP-FBG sensor, layer moduli, individual layer thickness, Poisson’s ratio, contact radial, and contact pressure, was employed for stress analysis. Notably, while the software mandates the input of contact pressure, this study did not delve into its determination process. This is because the input value of the contact pressure did not influence the outcome; it was solely used to calculate the stress factor from the software’s stress output. Regardless of the heightened stress output resulting from increased input values of the contact pressure, the stress factor, obtained by dividing stress by the contact pressure, remained constant when other input information remained unaltered.

To obtain the contact radius, tire information is essential. For this study, the tire specification was 275/80 R 22.5, where 275 represents the tire width in millimeters, 80 is the aspect ratio and equal to sidewall height divided by section width, “R” is for radial tires, and 22.5 is the rim diameter in inches (57.2 cm). A contact width of 75% of the tire section width is for optimum performance, as suggested by typical design criteria [42]. Therefore, for this tire, the contact width was 0.206 m, with the assumption that the contact width was 60% of the contact length [33], and the contact length was 0.344 m. And based on Equation (10), the contact area was 0.066 m^2^. With the software defining the contact area as a circle, the input contact radius was 0.145 m.

**Figure 9:**
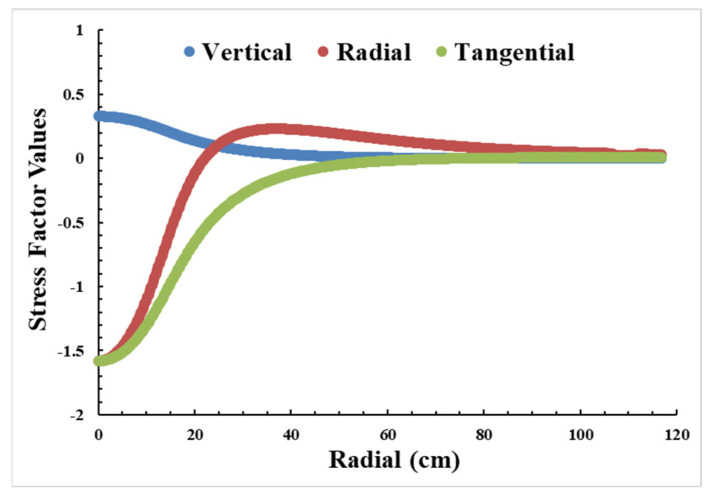
Vertical, longitudinal, and tangential stress factors.

The pavement information, as shown in Table 6 [33,40,43], the contact pressure, and the contact radius of the one wheel were input to the software KENPAVE to generate the stress when the distance between the center of the contact area and the sensor (installed 0.127 m under the road) was from 0 to 1.168 m. The software generated a comprehensive array of outputs, including vertical, radial, tangential, shear stress, and vertical displacement data, covering a radial coordinate range from 0 to 116.8 m. This research employed the vertical, radial, and tangential stress relative to weight to derive vertical, radial, and tangential factors (as depicted in Figure 9). Subsequently, these factors contributed to the utilization of Equation (9) for the determination of vehicle weight.

### 4.4. Accuracy Assessment of Vehicle Load Monitoring

**Figure 10:**
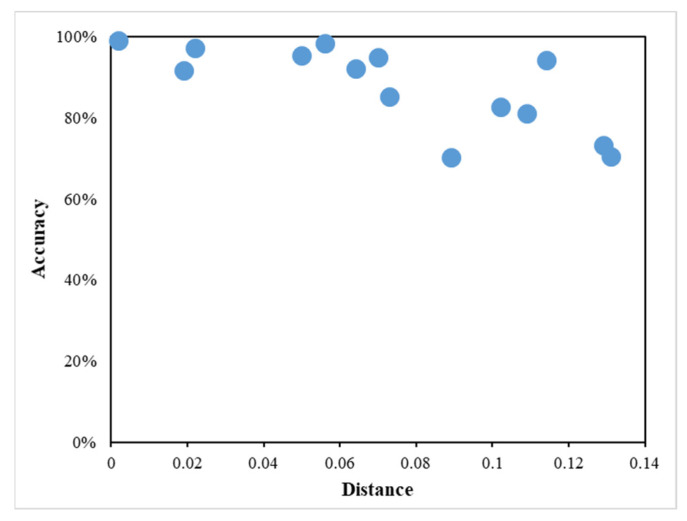
Vehicle measurement accuracy vs. distance between the embedded sensor and wheel loading position.

Having acquired the strain, distances between the sensors and wheel loading position, and the fitted stress factor to these distances, the calculation of the wheel load for the first right single wheel became feasible. Considering the same vehicle positioned differently during each drive, Table 7 provides the computed distances between the wheel and the sensor, calculated weight, and accuracy. The weight of the first right single wheel was assumed to be one-half of the weight of the first axle, which totals 26,022 N. This value was utilized as the actual weight of the first right single wheel for accuracy assessments. Generally, accuracy is closely linked to the distance between the wheel loading point and the sensors. For the FBG-1 sensor, the average accuracy reached 87.831% when the absolute distance from the FBG sensor to the wheel loading point was less than 0.089 m, decreasing to 84.206% when the distance fell below 0.131 m. Meanwhile, the FBG-2 sensor achieved an average accuracy of 94.645% when the sensor-to-wheel loading point distance was less than 0.070 m. When the distance is less than 0.109 m, the FBG-2 sensor will maintain an average accuracy of 91.027%. Figure 10 illustrates the alteration in vehicle measurement accuracy in response to changes in the distance between the two embedded GFRP-FBG sensors and the wheel loading position. As the distance between the sensors and the wheel loading position increases, there is a noticeable and consistent reduction in measurement accuracy. This observation underscores the importance of maintaining relatively close proximity between the sensors and the wheel loading point for optimal accuracy in vehicle weight measurement. Therefore, it is recommended to install the GFRP-FBG sensors approximately every 0.09 m and 0.07 m to achieve around 85% and 95% accuracy, respectively. Considering the significance of both driving speed and the distance between the wheel loading point and the FBG sensor, it is worth noting that this study primarily focuses on analyzing the distance’s impact on the results. This choice is driven by the practical challenge of controlling heavy trucks to drive at the same speed and in the same location. To validate the influence of speed on the measurement results, it is imperative to conduct additional runs with consistent speeds and controlled wheel loading positions. Further analysis of speed’s impact on measurement accuracy will be incorporated into future research.

## 5. Conclusions and Future Work

This study proposes an in-pavement sensor-camera hybrid system for wheel load detection on highways to assess the wandering effect of vehicles on the accuracy of WIM systems based on GFRP-FBG sensors. The hybrid system effectively addressed key challenges in WIM systems using either only FBG sensors or computer vision, including the lack of comprehensive research on the influence of vehicle wandering on WIM accuracy and the persistent difficulties in achieving accurate contact pressure assessment for vehicles using computer vision techniques. By integrating the spatial information from computer vision with strain assessments from GFRP-FBG sensors, an effective calibration approach was employed in this study to mitigate the wandering effect and enhance the accuracy of the WIM system based on FBG sensors. Key findings and conclusions from the study can be summarized as below:

1. Applying a calibration line to generate a model for distance prediction: This study employed distinct marked lines (L1, L2, L3, and L4) to develop a linear regression model accurately estimating the distance from the edge line to the wheel loading position. This model’s reliability is supported by a strong R-squared value of 0.9989 when the confidence interval is set at 95%, providing a solid basis for precise distance calculations;
2. Investigation of the wander effect: Given the fixed position of the sensor and the significant impact of varying wheel loading positions on the collected signals, this study accounted for the wandering effect using KENPAVE software. The outputs encompassed vertical, radial, and tangential stress, covering a sensor-wheel loading distance range of 0 to 116.8 m. Vertical, radial, and tangential factors were derived relative to weight, contributing to the determination of vehicle weight;
3. High-accuracy WIM measurement: Highly accurate measurement was achieved by taking into account factors such as the GFRP-FBG sensor-assessed strain, distance between the sensor and wheel loading position, and KENPAVE software-derived stress factors. Accuracy is closely tied to the proximity of the wheel loading point to sensors. FBG-1 achieves an average accuracy of 87.831% for distances under 0.089 m, decreasing to 84.206% when the distance is less than 0.131 m. In contrast, FBG-2 achieves 94.645% accuracy for distances less than 0.070 m and maintains 91.027% accuracy for distances under 0.109 m.

In conclusion, this study effectively validated the applicability of the proposed sensor-camera hybrid system for assessing single-wheel loads while the vehicle is in motion. Through a combination of using high-sensitive, strong, and durable GFRP-FBG sensors, precise calibration methods, and advanced software analyses, this paper provides valuable insights into weight evaluation techniques, particularly accounting for the wandering effect. The sensor-camera hybrid system’s innovative approach, which addresses the wandering effect, improves weight measurements for moving vehicles. This enhanced accuracy has practical applications in transportation infrastructure maintenance, traffic management, environmental impact assessment, pavement design, and research and development, contributing to safer roads and more efficient transportation systems. While this study primarily focuses on analyzing the distance’s impact on results due to practical challenges in controlling speed and location, it acknowledges the limitation of not fully exploring the impact of speed, truck load types and configuration, and installation layers within the pavement. Future research will address this limitation by conducting additional runs with controlled conditions to evaluate speed’s influence on measurement accuracy. Furthermore, while the study effectively evaluated single-wheel weights using the proposed methodology, future research avenues will include dual-wheel weight assessments. Analyzing load distribution and strain patterns in dual-wheel configurations could enhance our understanding of multi-axle vehicle behavior and its impact on pavement integrity, providing essential insights for both research and practical transportation scenarios.

## Figures and Tables

**Table 1:** Average dynamic modulus of elasticity value for test section [41].

Temp (°C)	25 Hz	10 Hz	5 Hz	1 Hz	0.5 Hz	0.1 Hz	0.05 Hz	0.01 Hz
−12	15.520	11.830	10.410	9.490	9.000	8.190	7.900	6.990
12	7.580	7.830	7.340	4.550	3.990	2.120	1.570	0.570
36	1.660	1.350	1.070	0.420	0.300	0.160	0.130	0.040

Note: unit is GPa.

**Table 2:** OFBG sensor strain transfer parameters.

	$E_{0}$ (GPa)	$r_{0}$ (mm)	$G_{GFRP}$ (GPa)	$r_{GFRP}$ (mm)	$r_{h}$ (mm)	$l_{f}$ (mm)
Longitudinal	70	0.0625	5.0	2.5	25	70

**Table 3:** Wavelength change data and calculated strain for 14 vehicle runs.

Vehicle Number	Wavelength Change (FBG-1)	Wavelength Change (T)	Strain (×10^−4^)	Vehicle Number	Wavelength Change (FBG-2)	Wavelength Change (T)	Strain (×10^−4^)
1	0.143	−0.002	1.194	8	0.143	0.001	1.186
2	0.132	−0.003	1.111	9	0.153	0.002	1.260
3	0.142	−0.002	1.183	10	0.127	0.000	1.057
4	0.151	−0.001	1.252	11	0.136	−0.002	1.153
5	0.105	−0.001	0.875	12	0.121	0.001	0.988
6	0.113	−0.002	0.951	13	0.126	−0.001	1.055
7	0.112	−0.001	0.931	14	0.082	−0.002	0.696

Note: unit of wavelength is nm.

**Table 4:** The length of the red and yellow calibration lines used to train the distance model.

Number	Red Calibration Line	Yellow Calibration Line (L1)	Yellow Calibration Line (L2)	Yellow Calibration Line (L3)	Yellow Calibration Line (L4)
1	0.030	0.114	0.102	0.089	0.073
2	0.032	0.113	0.100	0.087	0.072
3	0.033	0.112	0.099	0.086	0.071
4	0.035	0.111	0.098	0.085	0.069
5	0.037	0.110	0.097	0.084	0.068
6	0.038	0.109	0.096	0.083	0.067
7	0.040	0.108	0.095	0.082	0.066
8	0.041	0.106	0.093	0.080	0.065
9	0.043	0.105	0.092	0.079	0.064
10	0.045	0.104	0.091	0.078	0.063
11	0.046	0.103	0.090	0.077	0.062
12	0.048	0.102	0.089	0.076	0.061
13	0.049	0.101	0.087	0.074	0.060
14	0.051	0.100	0.086	0.073	0.059
15	0.052	0.099	0.085	0.072	0.058

Note: unit is m.

**Table 5:** Model-derived and adjusted wheel loading distances for 14 vehicle runs.

Vehicle Number	Distance (FBG-1)	Revised Distance (FBG-1)	Vehicle Number	Distance (FBG-2)	Revised Distance (FBG-2)
1	−0.125	−0.022	8	−0.101	0.002
2	−0.159	−0.056	9	−0.084	0.019
3	−0.176	−0.073	10	−0.053	0.050
4	−0.192	−0.089	11	−0.040	0.064
5	0.011	0.114	12	−0.033	0.070
6	−0.232	−0.129	13	−0.205	−0.102
7	0.027	0.131	14	−0.212	−0.109

Note: unit of distance is m.

**Table 6:** Parameters input to the software KENPAVE.

Layer	Thickness (m)	Poisson’s Ratio	Dynamic Modulus (GPa)
HMA	0.127	0.35	4.826
granular base	0.305	0.35	0.414
sub-base clay loam	0.305	0.35	0.276
granular	0.178	0.4	0.083
clay loam	0.089	0.4	0.083

**Table 7:** Vehicle load accuracy based on dynamic modulus of elasticity, KENPAVE stress factors, and GFRP-FBG strain evaluation.

Vehicle Number	Revised Distance (FBG-1)	Weight (FBG-1)	Accuracy (%)	Vehicle Number	Revised Distance (FBG-2)	Weight (FBG-2)	Accuracy (%)
1	−0.022	26,750.916	97.199	8	0.002	26,275.504	99.026
2	−0.056	26,429.119	98.435	9	0.019	28,168.385	91.752
3	−0.073	29,826.825	85.378	10	0.050	24,817.696	95.372
4	−0.089	33,747.883	70.310	11	0.064	28,061.367	92.163
5	0.114	27,505.864	94.298	12	0.070	24,698.723	94.915
6	−0.129	32,990.593	73.220	13	−0.102	30,495.575	82.808
7	0.131	33,672.318	70.601	14	−0.109	21,117.985	81.154

Note: unit of the distance and weight is m and N.

## Data Availability

The data presented in this study are available upon request from the corresponding authors.
